# SOCIAL DISCONNECTION, EPIGENETIC AGING, AND MOTORIC COGNITIVE RISK SYNDROME: A CAUSAL MEDIATION ANALYSIS

**DOI:** 10.1093/geroni/igad104.0323

**Published:** 2023-12-21

**Authors:** Xiang Qi, Daniel Belsky, Yang Yang, Susan Malone, Yaguang Zheng, Bei Wu

**Affiliations:** New York University, New York City, New York, United States; Columbia University, New York City, New York, United States; University of North Carolina at Chapel Hill, Chapel Hill, North Carolina, United States; New York University, New York City, New York, United States; New York University, New York City, New York, United States; New York University, New York City, New York, United States

## Abstract

Social isolation and loneliness are two different aspects of social disconnection. Motoric cognitive risk syndrome (MCR), a preclinical phase of dementia, is characterized by memory complaints and slow gait. It is unclear how social disconnection relates to MCR and its underlying mechanisms. Using data from Health and Retirement Study 2012-2020, we aimed to 1) examine the association between social isolation and loneliness and MCR and 2) investigate whether accelerated epigenetic aging mediates this association. We measured social isolation and loneliness at baseline (2012-2014) and epigenetic aging in 2016 using three DNA methylation-based measures (GrimAge, PhenoAge, and DunedinPoAm38), and recorded the incidence of MCR in 2016-2020. We used Cox proportional hazard models to investigate the association between social disconnection and MCR, and counterfactual mediation analysis to examine the role of epigenetic aging. Our sample consisted of 3,287 participants without MCR, with 678 developed MCR during follow-up. Our results showed that social isolation was significantly associated with an older GrimAge, a faster aging pace (DunedinPoAm38), and higher risks of MCR (HR, 1.36; 95% CI, 1.10-1.68), while loneliness was associated with faster DunedinPoAm38 aging pace but not MCR (HR, 0.95; 95% CI, 0.79-1.14). Further, GrimAge mediated 16% of the effect of social isolation (Indirect Effect HR, 1.09; 95% CI, 1.03-1.15), and DunedinPoAm38 mediated 11% (Indirect Effect HR, 1.06; 95% CI, 1.02-1.10). Findings suggest that epigenetic aging is a biological mechanism that underlies the association between social isolation and MCR. Further research is necessary to identify strategies to reduce social isolation for dementia prevention.

